# β-Elemene enhances susceptibility to cisplatin in resistant ovarian carcinoma cells via downregulation of ERCC-1 and XIAP and inactivation of JNK

**DOI:** 10.3892/ijo.2013.1996

**Published:** 2013-06-28

**Authors:** QUENTIN Q. LI, REBECCA X. LEE, HUASHENG LIANG, GANGDUO WANG, JUELI M. LI, YUHUA ZHONG, EDDIE REED

**Affiliations:** 1 Beihai Institute of Endocrine and Metabolic Diseases, Beihai, Guangxi 536000, P.R. China; 2 National Institutes of Health, Bethesda, MD 20892, USA; 3 West Virginia University School of Medicine, Morgantown, WV 26506, USA; 4 University of Maryland School of Pharmacy, Baltimore, MD 21201, USA

**Keywords:** apoptosis, cisplatin resistance, β-elemene, Chinese medicine, ovarian cancer, excision repair cross-complementation group-1, X-linked inhibitor of apoptosis protein, c-Jun NH_2_-terminal kinase

## Abstract

β-Elemene is a promising new plant-derived drug with broad-spectrum anticancer activity. It also increases cisplatin cytotoxicity and enhances cisplatin sensitivity in resistant human carcinoma cells. However, little is known about the mechanism of its action. To explore the potential therapeutic application of β-elemene as a drug-resistance modulator, this study investigated the underlying mechanism of β-elemene activity in cisplatin-resistant ovarian cancer cells. β-Elemene enhanced cisplatin sensitivity to a much greater extent in chemoresistant A2780/CP70 and MCAS human ovarian carcinoma cells compared to the chemosensitive parental cell line A2780. The dose-modifying factors for cisplatin were between 35 and 60 for A2780/CP70 cells and between 1.6 and 2.5 for A2780 cells. In the cisplatin-resistant ovarian carcinoma cells, β-elemene abrogated cisplatin-induced expression of excision repair cross-complementation group-1 (ERCC-1), a marker gene in the nucleotide excision repair pathway that repairs cisplatin-caused DNA damage. In addition, β-elemene not only reduced the level of X-linked inhibitor of apoptosis protein (XIAP), but also downregulated cisplatin-mediated XIAP expression in chemoresistant cells. Furthermore, β-elemene blocked the cisplatin-stimulated increase in the level of phosphorylated c-Jun NH_2_-terminal kinase (JNK) in these cells. These novel findings suggest that the β-elemene enhancement of cisplatin sensitivity in human chemoresistant ovarian cancer cells is mediated at least in part through the impairment of DNA repair activity and the activation of apoptotic signaling pathways, thereby making resistant ovarian cancer cells susceptible to cisplatin-induced cell death.

## Introduction

Human ovarian carcinoma remains a major cause of mortality and morbidity in the United States (1). Roughly 80% of patients will present with advanced-stage disease, but current chemotherapy is able to produce significant response rates and even long-term remission in only 20% of these women. *cis*-Diamminedichloroplatinum (II) (cisplatin) is one of the most effective anticancer drugs in the treatment of human ovarian cancer and other tumors (2–4). However, the efficacy of cisplatin is hampered because cancer cells acquire resistance to its cytotoxicity. Although the mechanism of cisplatin resistance *in vivo* is not clearly defined, laboratory studies with tumor tissues and cell lines suggest that enhanced nucleotide excision repair (NER) of cisplatin-caused DNA damage and impaired cisplatin-induced apoptosis play crucial roles in the development of the cisplatin-resistance phenotype (4–6). The expression of DNA repair genes such as excision repair cross-complementation group-1 (*ERCC-1*) and xeroderma pigmentosum complementation group A (*XPA*) is reported to be strongly associated with a poor prognosis in ovarian carcinoma and other tumors (7,8). Therefore, compounds that can circumvent cisplatin resistance and augment the effects of chemotherapy are needed.

**Figure 1:**
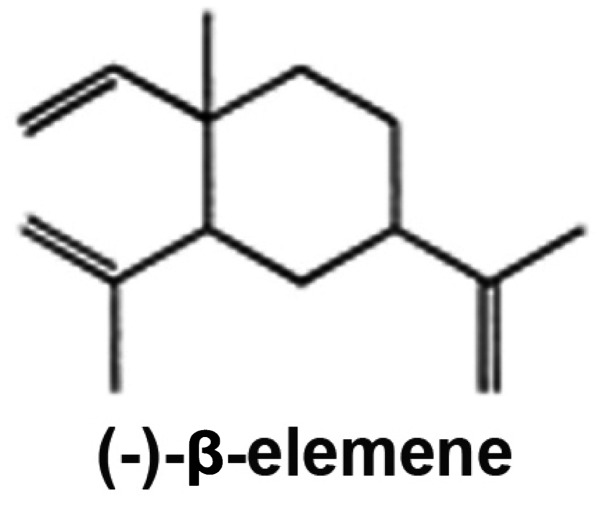
The chemical structure of β-elemene.

One candidate drug to fulfill this role is β-elemene (β-1-methyl-1-vinyl-2,4-di-isopropenyl-cyclohexane). β-Elemene (Fig. 1), a natural anticancer plant-derived drug, was approved by the Chinese Food and Drug Administration for the treatment of human cancers. The major advantages of β-elemene as an anticancer drug are: i) it has broad-spectrum antitumor effects in many types of cancer, including drug-resistant tumors; ii) it does not direct multidrug resistance and can reverse the resistance to other drugs; and iii) it has low toxicity and is therefore well tolerated and accepted by patients with cancer. The effect of β-elemene on other drug sensitivity in human tumors is unknown. We have recently reported that β-elemene increases sensitivity to cisplatin and augments cisplatin-induced apoptosis in chemoresistant human ovarian cancer cells and other tumor cells (9–20). These novel findings indicate that β-elemene may be efficacious in the treatment of cisplatin-resistant tumors.

We hypothesized that β-elemene enhancement of cisplatin sensitivity in human chemoresistant ovarian cancer cells is mediated at least in part through the regulation of DNA repair activity and apoptotic death signaling. To test this hypothesis and elucidate the mechanism underlying the effect of β-elemene on cisplatin cytotoxicity, we investigated whether β-elemene promotes apoptotic cell death in cisplatin-treated resistant ovarian cancer cells by downregulating the expression of ERCC-1 and X-linked inhibitor of apoptosis protein (XIAP) and inactivating c-Jun NH_2_-terminal kinase (JNK). This research is significant because β-elemene may be useful therapeutically as a modulator of platinum drug resistance in human malignancies. Our results provide a framework for exploiting the combination of β-elemene and cisplatin as a potentially effective chemotherapy regimen to overcome cisplatin drug resistance in ovarian cancer and other tumors.

## Materials and methods

### Chemicals and immunoreagents

The (−)-β-elemene (98% purity) was obtained from Yuanda Pharmaceuticals Ltd. Inc. (Dalian, China). Cisplatin, dimethylsufoxide and other chemicals were purchased from Sigma-Aldrich Chemical Co. (St. Louis, MO, USA). Antibodies against XIAP and β-actin, peroxidase-labeled anti-rabbit immunoglobulin G (IgG), Blotto B, and the ECL western blot analysis system were purchased from Santa Cruz Biotechnology Inc. (Santa Cruz, CA, USA). Anti-phospho-JNK1 (Thr183/Tyr185) antibody was purchased from Cell Signaling Technology (Beverly, MA, USA), as described previously (21,22).

### Cells and cell culture conditions

The human cisplatin-resistant ovarian cancer cell lines A2780/CP70 and MCAS and the parental sensitive ovarian cancer cell line A2780 have been studied extensively by our laboratory (15,17,18). The cells were cultured in monolayers using RPMI-1640 medium (Invitrogen, Life Technologies, Gaithersburg, MD, USA) supplemented with 10% (v/v) fetal bovine serum, 50 U/ml penicillin, and 50 μg/ml streptomycin (Invitrogen), and were grown in logarithmic growth at 37°C in a humidified atmosphere of 5% CO_2_ and 95% air. The cells were routinely tested for mycoplasma infection using a commercial assay system (MytoTect; Invitrogen), and new cultures were established monthly from frozen stocks. All media and reagents contained <0.1 ng/ml endotoxin as determined by a *Limulus polyphemus* amebocyte lysate assay (Whittaker Bioproducts, Walkersville, MD, USA). Before starting the experiments, the cells were sub-cultured and grown to 70–80% confluence. Cisplatin was initially dissolved at 5 mM in phosphate-buffered saline (PBS) without Ca^2+^ or Mg^2+^. Cisplatin and β-elemene were serially diluted, respectively, in culture medium to obtain the desired concentrations.

### Cell growth inhibition assay

The antiproliferative effects of β-elemene alone, cisplatin alone, and cisplatin plus β-elemene were assessed using the 3-(4,5-dimethylthiazol-2-yl)-2,5-diphenyltetrazolium bromide (MTT) assay (Promega Corp., Madison, WI, USA) according to the manufacturer’s instructions. In brief, the cells were evenly distributed in 96-well plates (5×10^3^ cells/well), grown overnight and then treated for 24, 48, 72 and 96 h with β-elemene alone (0, 20, 40, 60, 80, 100, 120, 140, 160, 180 and 200 μg/ml), cisplatin alone (0, 1.0, 2.0, 4.0, 8.0, 16.0, 32.0, 64.0, 128.0, 256.0 and 512.0 μM), or a combination of cisplatin (at the above concentrations) plus β-elemene (40 μg/ml). After incubation, 20 μl of CellTiter 96 Aqueous One Solution reagent were added to each well of the assay plates containing treated and untreated cells in 100 μl of culture medium, and the plates were incubated at 37°C and 5% CO_2_ for 1–4 h. The optical density at 590 nm was determined using a 96-well Opsys MR™ microplate reader (Thermo Labsystems, Chantilly, VA, USA). Proliferation rates were calculated from the optical density of drug-treated cells relative to that of cells with no added drug (control value, 100%), as follows: percentage cell viability = [(OD with drug - blank) ÷ (OD without drug - blank)] × 100. The half-maximal inhibitory concentration (IC_50_) was determined from the dose-response curves. The dose-modifying factor (DMF) was calculated as the IC_50_ for cisplatin without β-elemene divided by the IC_50_ for cisplatin with β-elemene: DMF = IC_50_ (cisplatin) ÷ IC_50_ (cisplatin + β-elemene).

### Generation of ERCC-1 antiserum

Polyclonal anti-peptide antiserum was generated by Bio-Synthesis Inc. (Lewisville, TX, USA). A synthetic peptide containing the carboxy-terminus of ERCC-1 was coupled to keyhole limpet hemocyanin using m-maleimidobenzoyl-N-hydroxysuccinimide ester as a cross-linker. This was used to immunize New Zealand white female rabbits, which were bled at regular intervals to obtain serum containing the antibodies. The undiluted antiserum was used in western blot analyses, as described previously (23,24).

### Protein extraction and western blot analysis

Ovarian tumor cells treated with β-elemene, cisplatin or their combination were harvested by trypsinization, washed with ice-cold PBS, and lysed on ice for 30 min in mammalian cell lysis buffer (Quality Biological Inc., Gaithersburg, MD, USA) containing 10 μl/ml 200 mM phenylmethylsulfonyl fluoride, 10 μl/ml 100 mM sodium orthovanadate and 10 μg/ml aprotinin. Lysates were clarified by centrifugation at 13,000 × g for 30 min at 4°C, and the protein concentrations in the supernatants were determined by Bradford assay (Bio-Rad, Richmond, CA, USA). Proteins (40 to 60 μg) from whole-cell lysates were mixed 1:1 with 2X sodium dodecyl sulfate (SDS) gel solution (Quality Biological Inc.), heated for 5 min at 95°C, separated by 10% SDS-polyacrylamide gel electrophoresis, and transferred to nitrocellulose membranes (Schleicher & Schuell BioScience Inc., Keene, NH, USA). After blocking in Blotto B for 1 h at room temperature, the membranes were incubated overnight at 4°C with specific primary antibodies (diluted 1:100–1:300). The membranes were washed with TBS/0.1% Tween-20 solution, incubated with anti-rabbit peroxidase-conjugated secondary antibody (diluted 1:10,000), and washed again. Immunoreactive bands were detected with enhanced chemiluminescence substrate according to the manufacturer’s instructions and visualized using X-ray film (Eastman Kodak, Rochester, NY, USA). All blots shown are representative of three independent experiments.

### Statistical analysis

All quantitative values are presented as means ± SD. Data were analyzed using two-way analysis of variance (ANOVA) for comparison among groups. Student’s *t*-test was used to analyze the significance of differences between untreated and treated groups. All p-values were determined using a two-sided *t*-test, and p-values <0.05 were considered to indicate significance.

## Results

### β-Elemene suppresses cell proliferation and augments cisplatin-induced cytotoxicity in resistant and sensitive human ovarian cancer cells

We first examined the *in vitro* antitumor activity of β-elemene in human ovarian carcinoma cells, as determined by the MTT assay. β-Elemene at concentrations of 20–200 μg/ml dose-dependently inhibited the growth and proliferation of both A2780 and A2780/CP70 cells at 24, 48, 72 and 96 h, with IC_50_ values at 24, 48, 72 and 96 h ranging between 60 and 65 μg/ml for A2780 cells and between 65 and 80 μg/ml for A2780/CP70 cells (Table I). Similarly, the IC_50_ values of β-elemene for MCAS cells were between 60 and 78 μg/ml. The IC_50_ values were not significantly different between the cisplatin-sensitive (A2780) and cisplatin-resistant (A2780/CP70 and MCAS) cell lines (p>0.05), indicating that β-elemene has a similar antitumor activity toward both sensitive and resistant ovarian cancer cells *in vitro*. Thus, cisplatin-resistant ovarian tumor cells are still sensitive to β-elemene.

Next, we assessed the enhancing effect of β-elemene on cisplatin cytotoxicity in human ovarian tumor cells using the MTT assay. A2780 and A2780/CP70 cells were exposed to cisplatin alone or in combination with β-elemene (40 μl/ml) for 24, 48, 72 and 96 h, and the inhibition of cell growth was measured *in vitro*. At concentrations of 0 to 512.0 μM, cisplatin caused a dose-dependent inhibition of A2780 and A2780/CP70 cell proliferation at all four time points. The IC_50_ values of cisplatin alone for chemoresistant A2780/CP70 cells at 24, 48, 72 and 96 h were 95.0, 66.0, 65.0, and 60.0 μM, respectively, and decreased strikingly to 2.5, 1.9, 1.85 and 1.0 μM, respectively, when cisplatin was combined with β-elemene (Table I; p<0.01); the dose-modifying factors (DMFs) for cisplatin in A2780/CP70 cells ranged from 35 to 60. Similarly, the IC_50_ of cisplatin alone for MCAS cells was 38.0 μM and was reduced markedly to 6.5 μM when cisplatin was combined with β-elemene (p<0.01); the DMF in this cell line was 5.85. Although the IC_50_ values of cisplatin alone for chemosensitive A2780 cells (6.2, 1.75, 1.6 and 1.5 μM at 24, 48, 72 and 96 h, respectively) decreased significantly when cisplatin was combined with β-elemene (3.8, 0.8, 0.75 and 0.6 μM, respectively) (Table I; p<0.05), the DMFs for cisplatin in A2780 cells, which ranged from 1.6 to 2.5, were significantly lower than those in A2780/CP70 and MCAS cells (p<0.05). These results suggest that β-elemene and cisplatin may act synergistically to enhance cytotoxicity in both chemoresistant and chemosensitive ovarian carcinoma cells, but that β-elemene has a greater effect on cisplatin sensitivity in chemoresistant ovarian tumor cells.

### The effect of β-elemene on the protein level of ERCC-1, phospho-JNK1 and XIAP in chemoresistant human ovarian carcinoma cells

**Figure 2:**
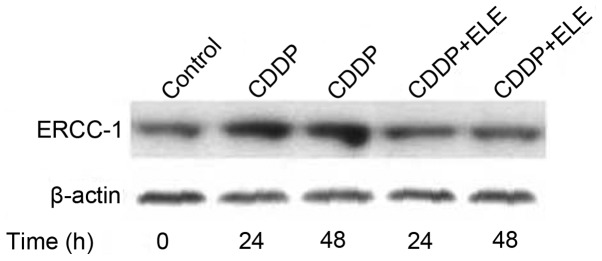
β-Elemene blocks the cisplatin-induced increase in ERCC-1 protein in human ovarian tumor cells. A2780/CP70 cells were exposed to 40 μM cisplatin (CDDP) alone or cisplatin plus β-elemene (ELE) at the IC_20_ for 24 or 48 h. The cells were harvested and lysed. Cell lysates containing 40 μg of protein were analyzed on western blots using ERCC-1 antiserum and antibody against β-actin (loading control), as described previously (23,24).

Enhanced DNA repair capacity contributes to cisplatin resistance in human tumors, and NER is responsible for the repair of platinum-DNA adducts in human cells. Furthermore, high levels of ERCC-1 protein support the efficient DNA repair capacity of resistant cancer cells and *ERCC-1* is a marker gene for the NER mechanism. Thus, to investigate whether the mechanism by which β-elemene reverses drug resistance to cisplatin involves, at least in part, the inhibition of DNA repair activity, we tested the effect of β-elemene on cisplatin-upregulated ERCC-1 expression in resistant ovarian cancer cells. β-Elemene significantly attenuated the cisplatin-induced increase in the ERCC-1 protein level in A2780/CP70 ovarian cancer cells (Fig. 2), indicating that the reduction of DNA repair activity by β-elemene is positively associated with increased cisplatin cytotoxicity in resistant ovarian tumor cells.

**Figure 3:**
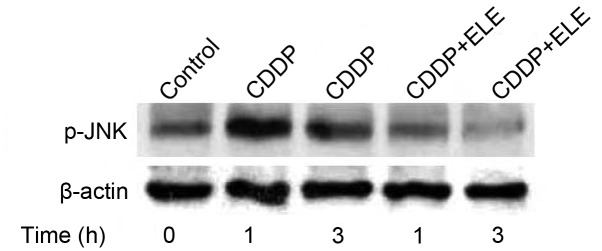
β-Elemene abrogates cisplatin-induced JNK1 protein phosphorylation in human ovarian tumor cells. A2780/CP70 cells were exposed to 40 μM cisplatin alone or cisplatin plus β-elemene (ELE) at the IC_20_ for 1 or 3 h. The cells were harvested and lysed. Cell lysates containing 60 μg of protein were analyzed on western blots using antibodies against phospho-JNK1 (Thr183/Tyr185) and β-actin (loading control), as described previously (21,22).

The upregulation of *ERCC-1* gene expression is mediated by activator protein 1 (AP-1) transcriptional activity, which is activated by a JNK phosphorylation cascade. In A2780/CP70 cells, β-elemene inhibited the cisplatin-induced increase in JNK phosphorylation (Fig. 3), suggesting that β-elemene may suppress ERCC-1 expression via a phosphatidylinositol 3-kinase (PI3K)/JNK signaling pathway.

**Figure 4:**
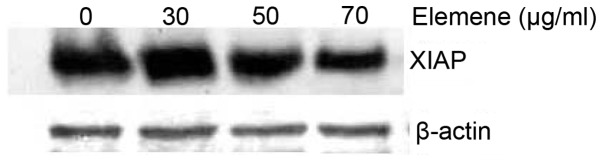
β-Elemene downregulates XIAP protein levels in human ovarian tumor cells. A2780/CP70 cells were exposed to β-elemene at the indicated concentrations for 24 h. The cells were harvested and lysed. Cell lysates containing 60 μg of protein were analyzed on western blots using antibodies against XIAP and β-actin (loading control), as described previously (16).

**Figure 5:**
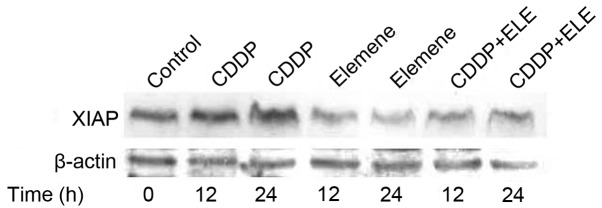
The effect of β-elemene and cisplatin on XIAP protein levels in human ovarian tumor cells. A2780/CP70 cells were exposed to 30 μM cisplatin (CDDP) alone, 70 μg/ml β-elemene alone, or cisplatin plus β-elemene (ELE) at the IC_20_ for 12 or 24 h. The cells were harvested and lysed. Cell lysates containing 40 μg of protein were analyzed on western blots using antibodies against XIAP and β-actin (loading control).

Activation of the PI3K/protein kinase B (Akt) pathway leads to the upregulated expression of XIAP, which modulates death signaling pathways and is a determinant of cisplatin resistance in ovarian cancer cells. β-Elemene not only reduced the XIAP protein level (Fig. 4) but also abrogated cisplatin-induced XIAP expression in resistant ovarian tumor cells (Fig. 5), indicating the involvement of XIAP in the mechanism of β-elemene action in resistant ovarian cancer cells.

## Discussion

The greatest limitation to the successful treatment of ovarian cancer is the development of clinical resistance to cisplatin. Although the mechanism of cisplatin resistance *in vivo* is not clearly understood, laboratory studies on tumor tissues and cell lines suggest that resistance to cisplatin is multifactorial (25,26). These factors include impaired cellular uptake of cisplatin (27), increased intracellular detoxification by glutathione and metallothionein systems (28), altered patterns of DNA platination, impaired cisplatin-induced apoptosis and enhanced repair of platinum-damaged DNA (4–8,26,27,29,30).

We have been investigating the mechanisms of cisplatin drug resistance, focusing on the relationship between DNA repair and cisplatin resistance in ovarian cancer and other tumors (21–24,31–36). Increasing evidence indicates that NER is responsible for the repair of platinum-caused DNA damage (4–8,37). Repair-defective cells are hypersensitive to cisplatin (38,39), and enhanced DNA repair has been implicated in the cisplatin-resistance phenotype (27,30,40). Furthermore, increased repair of cisplatin-caused interstrand cross-links and intrastrand adducts is associated with resistance in human ovarian cancer cells (41) and in laboratory-derived cisplatin-resistant lines (27). ERCC-1 is a key DNA repair protein in the NER process and a useful biomarker for NER activity in human cells. The overexpression of *ERCC-1* and other NER genes has been associated with the repair of cisplatin-induced DNA damage (30,37,41) and clinical resistance to cisplatin (42,43). The expression levels of *ERCC-1* in cisplatin hypersensitive, repair-deficient cells are 50- to 30-fold lower than those in resistant cells. These observations indicate that enhanced DNA repair capacity contributes to the development of cisplatin resistance in human cancers. In the present study, we showed that β-elemene increased the sensitivity to cisplatin and blocked cisplatin-induced ERCC-1 protein expression in resistant human ovarian cancer cells, suggesting that β-elemene enhances cisplatin sensitivity in resistant ovarian cancer cells by decreasing the proficiency of repair of cisplatin-induced DNA damage.

A number of studies have reported that NER gene expression is regulated by the JNK/AP-1 pathway in response to cisplatin *in vitro*. The AP-1 family of transcription factors is a heterodimeric protein composed of proteins belonging to the c-Fos, c-Jun, ATF and JDP families, and is responsible for the activation of a wide variety of genes in different cell types and tissues. AP-1 binding sites (5′-TGAG/CTCA-3′) are frequently found in promoters or enhancers of genes that are inducible by cisplatin. Evidence has shown that cisplatin induces the expression of *c-fos/c-jun*(21,22,32) and activates JNK (21,22,32,44,45) in ovarian cancer cells. Therefore, the activation of AP-1 and subsequent overexpression of AP-1-regulated NER genes may enhance DNA repair capacity in affected cells and contribute to decreased chemosensitivity in human ovarian cancer cells (37,41).

This hypothesis is supported by several lines of evidence. We have previously demonstrated that cisplatin exposure activates an AP-1-mediated increase in *ERCC-1* expression in human ovarian tumor cells (23,32,33). Treatment with phorbol ester, an AP-1 agonist, also induced increases in ERCC-1 mRNA and protein levels in human ovarian carcinoma cells *in vitro*(23,24,34). AP-1 may be a common activator of NER genes (46). The overexpression of wild-type c-Jun is associated with cisplatin resistance (44). In contrast, the inhibition of AP-1 activity in cells modified by inhibition of Gli1 with a specific short-hairpin RNA downregulates c-Jun activity and NER gene (*ERCC-1* and *XPD*) expression, blocks platinum-DNA adduct repair, and results in supra-additive cell killing with cisplatin (44,47). Furthermore, cells in which AP-1 has been genetically inactivated are hypersensitive to genotoxic insults, including anticancer agents. These findings suggest that AP-1 may play a prominent role in modulating DNA repair processes in both physiological and pathophysiological conditions. AP-1-dependent DNA repair activities may provide a stress-protective function in cells by effectively reducing the cytotoxic, mutagenic, and carcinogenic consequences of DNA damage. This may also explain, at least in part, the observed increase in mRNA levels of *ERCC-1* and other NER genes in clinical platinum-resistant specimens (6,42,43,48).

Given that the promoters of *ERCC-1* and other NER repair genes contain AP-1 binding sites, signal transduction pathways that modulate AP-1 may be important in the regulation of DNA repair. JNK, a member of the MAP kinase family and Ras pathway, is responsible for the phosphorylation of c-Jun protein at serine residues 63 and 73 in the NH_2_-terminal domain, which results in greatly enhanced AP-1 binding to regulated genes and subsequent transcriptional regulation (49). Recent work has shown that cellular damage induced by DNA damaging agents, including cisplatin (44,50), activates the JNK pathway involving AP-1. This response has been reported to protect against cisplatin-induced DNA damage by allowing DNA repair and survival following cisplatin treatment (44). Inhibition of this pathway in cells modified by the overexpression of a dominant-negative mutant of c-Jun blocks DNA repair and leads to decreased viability following treatment with cisplatin (44). These observations suggest that the Ras/JNK pathway may mediate a physiological response to DNA damage. We have previously shown that upon stimulation with cisplatin, JNK may directly phosphorylate c-Jun at serine residues 63 and 73 to activate *ERCC-1* transcription via AP-1 in A2780/CP70 ovarian cancer cells (32), which would place *ERCC-1* under the influence of the JNK/Ras/AP-1 signal transduction pathway. However, the upstream signaling cascade leading to the activation of JNK in response to cisplatin-induced DNA damage remains to be further elucidated.

PI3K is a heterodimer composed of one regulatory subunit (p85) and one catalytic subunit (p110) (51). The catalytic subunit of PI3K phosphorylates phosphatidylinositol (PI) at the 3′ position of the inositol sugar ring, generating PI 3-phosphate, PI 3,4-bisphosphate, and PI 3,4,5-trisphosphate (51). Experiments with PI3K inhibitors, constitutively active PI3K mutants, and dominant-negative PI3K mutants have established an important role for PI3K in apoptosis (51,52). The best-known downstream target of PI3K is the serine-threonine kinase Akt, which transmits survival signals from growth factors (53). However, PI3K also has many other targets, including NF-κB (54), BAD (55) and JNK (56), and is involved in cell growth, proliferation, differentiation and survival. Therefore, β-elemene may affect DNA repair activity in these cells by regulating the PI3K/JNK/AP-1 signaling pathway, leading to the downregulated expression of *ERCC-1* and other NER genes, and cell death.

**Figure 6:**
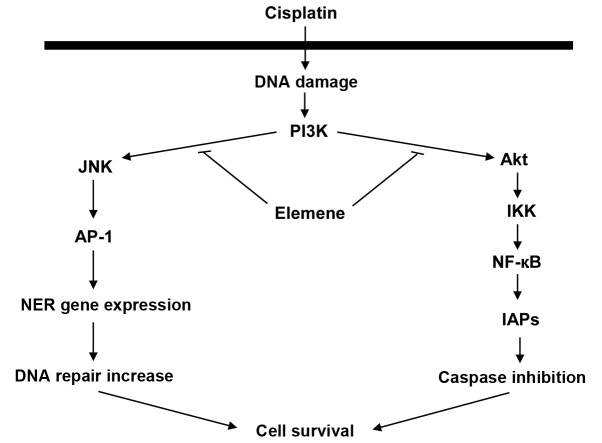
Proposed molecular mechanism for the effect of β-elemene on cisplatin chemosensitivity in human ovarian carcinoma cells. In the proposed mechanism shown here, β-elemene mediates altered DNA repair activity and cisplatin sensitivity in human ovarian carcinoma cells by blocking cisplatin-induced PI3K/JNK and PI3K/Akt activation, thereby preventing from the activation of the downstream signaling components such as AP-1 and NF-κB. This leads to decreased DNA-repair activity and increased caspase activity, respectively, which both confer sensitization of resistant ovarian cancer cells to the chemotherapeutic agent cisplatin. See text for details.

A proposed mechanism that is consistent with the evidence is presented in Fig. 6. In this model, cisplatin increases PI3K activity, which activates JNK, and JNK activates AP-1. Activated AP-1 upregulates NER gene expression, thereby increasing DNA repair activity and cell survival. This mechanism may be responsible for reduced cellular sensitivity to cisplatin. β-Elemene may sensitize resistant ovarian cancer cells to cisplatin by blocking the activation of the PI3K/JNK signaling pathway, which would reduce DNA repair activity and enhance cisplatin cytotoxicity. In our previous study, cisplatin increased Raf-1 and c-Fos expression in human ovarian carcinoma cells (22). PI3K and Akt regulate the effect of Raf on gene expression. Therefore, there are also three alternative PI3K/Akt/Raf signaling pathways that may link cisplatin to AP-1 activation. In all three pathways, cisplatin-activated PI3K acts through a phosphorylation cascade to activate Akt and Raf, which can then trigger three different pathways to activate AP-1: i) Raf → MEKK1 → MKK4 → JNK → c-Jun; ii) Raf → MEK1/2 → ERK1/2 → c-Jun; and iii) MEK1/2 → ERK1/2 → p90^RSK^ → CREB → c-Fos. Phosphorylated c-Jun and/or c-Fos then augment AP-1 activity, which results in upregulated NER gene expression, enhanced DNA repair capacity and increased cell survival.

The major goal of cancer chemotherapy is to commit tumor cells to death or apoptosis following exposure to anticancer agents. Considerable evidence collected during the past decades indicates that cisplatin kills cells through the induction of cell apoptosis (57). The mechanisms of cisplatin-induced apoptosis are complex and involve many regulators (58). Caspase cascades are activated in response to cisplatin exposure, and this activation leads to an irreversible commitment to apoptotic cell death (25,59,60). Caspases are held in check, in part, by protein-protein interactions with inhibitor of apoptosis proteins (IAPs). The IAPs such as XIAP, cellular IAP-1, and cellular IAP-2 bind directly to caspases such as caspase-3, -7 and -9, and inhibit their enzymatic activities. In mammalian cells, two major regulatory pathways have been proposed for caspase cascades, an extrinsic pathway and an intrinsic pathway (61).

In the extrinsic pathway, the Fas receptor is activated by the binding of an extracellular ligand such as Fas ligand (FasL), and this induces the assembly of a death-inducing signaling complex, which includes the Fas-associated death domain protein as an adaptor and procaspase-8 (or procaspase-10). The procaspase is activated and in turn initiates the activation of two effector caspases, caspase-3 and -7 (62). The Fas/FasL-activated caspase-8/caspase-3 pathway may be involved in tumor cell response to cisplatin (58,63–65). Alterations in this apoptotic signaling pathway, such as defects in the expression of CD95L or CD95, or defects in caspase-8 or caspase-3 activation, may contribute to cisplatin-resistance. Conversely, increased caspase levels may restore sensitivity to cisplatin chemotherapy in tumor cells (64,65). The intrinsic pathway of apoptosis is mediated by members of the Bcl-2 family, which destabilize the mitochondrial membrane, causing the release of cytochrome *c* from mitochondria. In the presence of ATP and cytochrome *c*, apoptotic protease-activating factor-1 (Apaf-1) activates caspase-9, which in turn activates caspase-3 (62,66). Although the exact mechanism whereby Bcl-2 family members regulate mitochondrial damage remains under debate, Bcl-2 and Bcl-X_L_ are thought to exert anti-apoptotic effects by stabilizing the mitochondrial membrane potential and preventing the release of apoptosis-inducing molecules such as cytochrome *c*(66–68). Cisplatin may cause mitochondrial release of cytochrome *c* and activation of caspase-3 by acting through Bcl-2 family proteins (59,69). Cisplatin has been shown to induce the expression of Bax and/or the cleavage of Bcl-2 to increase the Bax:Bcl-2 ratio and activate the apoptotic cascade (59,70). Moreover, antisense oligonucleotides targeting Bcl-2 and Bcl-X_L_ sensitized tumor cells to the cytostatic effect of cisplatin (68).

Failure of the apoptotic pathways may lead to cisplatin resistance. Recent evidence has demonstrated that the failure to upregulate FasL in response to cisplatin exposure is associated with chemoresistance in ovarian cancer cells (71). In one study, cisplatin decreases the XIAP content in cisplatin-sensitive, but not cisplatin-resistant, human ovarian cancer cells (72). Other researchers have confirmed this finding, showing that the acquisition of cisplatin resistance is associated with the ability of cisplatin-treated ovarian tumor cells to upregulate XIAP expression (73). These observations indicate that impaired cisplatin-induced apoptosis may account for the chemoresistance to cisplatin therapy in ovarian tumors.

In the present study, β-elemene suppressed XIAP expression and blocked cisplatin-induced XIAP upregulation in resistant ovarian tumor cells. PI3K activates Akt and NF-κB, which block apoptosis by upregulating the expression of IAP family of proteins, thereby inhibiting the activities of caspase-3, -7 and -9. Thus, β-elemene may enhance cisplatin sensitivity in resistant ovarian cancer cells by abrogating cisplatin-induced PI3K activity and the PI3K/Akt signaling pathway, resulting in decreased IAP expression and increased apoptotic cell death. In a proposed mechanism that is consistent with these findings, cisplatin-induced activation of the PI3K/Akt pathway causes the phosphorylation of IKKα and subsequent activation of the transcription factor NF-κB, which upregulates IAP expression to inhibit caspase activation and promote cell survival: cisplatin → PI3K → Akt → IKKα → NF-κB → IAP expression → caspase inhibition → cell survival (Fig. 6). Our previous demonstration of cisplatin-enhanced Raf-1 activity in human ovarian cancer cells (22) suggests that IAP expression may also be upregulated by two alternative signaling pathways involving Raf: i) cisplatin → Ras → PI3K → PDK → Akt → Raf → MEKK1 → IKKα → NF-κB → IAP expression; and ii) cisplatin → Ras → Raf → MEKK1 → IKKα → NF-κB → IAP expression. This presents the possibility of crosstalk between the cisplatin-activated PI3K/PDK/Akt/IKK signal transduction pathway and the cisplatin-activated Ras/Raf/MEKK1/IKK signaling pathway in the activation of NF-κB and IAP expression, and the inhibition of caspase activity. These pathways collaboratively lead to cell survival in cisplatin-resistant ovarian cancer cells.

On the basis of the evidence obtained in our studies and those of other groups, we propose that cisplatin increases PI3K activity, which increases JNK and AP-1 activation; activated AP-1 upregulates NER gene expression and increases DNA repair activity. This mechanism may reduce cellular sensitivity to cisplatin. In the current study, β-elemene promoted cisplatin cytotoxicity and apoptosis by blocking cisplatin-induced increases in the levels of ERCC-1 and XIAP in resistant ovarian tumor cells. Based on these observations, we propose that β-elemene alters DNA repair activity and cisplatin sensitivity in human ovarian carcinoma cells by preventing the cisplatin-induced activation of PI3K/JNK and PI3K/Akt, consequently blocking the activation of the downstream transcription factors AP-1 and NF-κB. This results in reduced DNA repair activity, enhanced caspase activity, and increased apoptotic cell death in cisplatin-resistant ovarian cancer cells. Fig. 6 illustrates the proposed signaling pathways responsible for chemotherapeutic resistance to cisplatin and a possible mechanism by which β-elemene may act as a drug-resistance modulator to enhance the antitumor activity of cisplatin in resistant human ovarian cancer cells.

Taken altogether, we showed in this study that β-elemene increases sensitivity to cisplatin; decreases cisplatin-induced expression of *ERCC-1*, a DNA repair gene, through blocking a JNK/AP-1 pathway; and augments cisplatin-induced cell death by downregulating XIAP expression in resistant ovarian cancer cells. These results suggest that β-elemene enhances susceptibility to cisplatin in resistant tumor cells through the regulation of DNA repair activity and apoptotic death signaling in human ovarian cancer. These novel findings indicate that β-elemene may be efficacious as a drug-resistance modulator for cisplatin-resistant carcinomas. This information provides a better understanding of the mechanisms of modulation of cisplatin sensitivity and assists in the design of potentially effective β-elemene-based chemotherapy regimens to overcome cisplatin resistance in human ovarian cancer and other tumor types.

## Figures and Tables

**Table I:** β-Elemene increases cisplatin cytotoxicity and enhances cisplatin sensitivity in human ovarian carcinoma cells, as determined by MTT assay.

	IC_50_			
	
Drug	24 h	48 h	72 h	96 h
A2780 cells				
β-Elemene (μg/ml)	65	65	65	60
Cisplatin (μM)	6.2	1.75	1.6	1.5
β-Elemene + cisplatin (μM)	3.8	0.8	0.75	0.6
Dose-modifying factor	1.6	2.2	2.1	2.5
A2780/CP70 cells				
β-Elemene (μg/ml)	80	70	68	65
Cisplatin (μM)	95	66	65	60
β-Elemene + cisplatin (μM)	2.5	1.9	1.85	1.0
Dose-modifying factor	38	34.7	35.1	60

The results reveal that β-elemene increased cisplatin cytotoxicity by 34.7- to 60-fold in A2780/CP70 cells, but only 1.6- to 2.5-fold in A2780 cells. The IC_50_ (half maximal inhibitory concentration) value is defined as the concentration of β-elemene or cisplatin needed for 50% inhibition of cell growth and proliferation. The dose-modifying factor (DMF) was calculated as the IC_50_ for cisplatin without β-elemene divided by the IC_50_ for cisplatin with β-elemene: DMF = IC_50_ (cisplatin) ÷ IC_50_ (cisplatin + β-elemene). MTT, 3-(4,5-dimethylthiazol-2-yl)-2,5-diphenyltetrazolium bromide.
